# The association between body mass index elevation and differentiation in vitamin D receptor gene expression, genetic polymorphism, and oxidative stress in adult Egyptian individuals

**DOI:** 10.1038/s41598-023-44607-4

**Published:** 2023-10-17

**Authors:** Nadia Z. Shaban, Mai Abdel-Alnaby, Mohamed N. Atta, Ahmed A. Abdul-Aziz, Fayed Megahed

**Affiliations:** 1 Biochemistry Department, Faculty of Science, Alexandria University, Alexandria, 21511 Egypt; 2 Endocrinology Unit, Department of Internal Medicine, Faculty of Medicine, Alexandria University, Alexandria, Egypt; 3 Nucleic Acid Research Department, Genetic Engineering and Biotechnological Research Institute, City of Scientific Research and Technological Applications (SRTA-City), Alexandria, Egypt

**Keywords:** Biochemistry, Cell biology, Genetics, Molecular biology, Biomarkers, Diseases, Endocrinology, Health care, Medical research, Molecular medicine, Risk factors

## Abstract

Vitamin D plays a central role in maintaining calcium, phosphorus, and bone homeostasis in close interaction with the parathyroid hormone. Obesity is a significant health problem worldwide, particularly in developed nations. The current study was carried out to investigate the possible relationship between body mass index (BMI) elevation and differentiation in 25-hydroxyvitamin D (VD), vitamin D receptor (VDR) gene expression, and genetic polymorphism besides oxidative stress in adult Egyptian individuals. This was done to explore the mechanisms underlying the suggested role of the VD/VDR complex in the pathogenesis of obesity. A total of 70 subjects (30 obese, 25 overweight, and 15 normal, age: 20–50 years, without other chronic diseases) were selected. The study focused on the determination of VD, VDR gene polymorphism, VDR gene expression, alkaline phosphatase, calcium, phosphorus, glucose, lipid profile, oxidative stress including, oxidant (malondialdehyde), and anti-oxidants (reduced glutathione and superoxide dismutase). The results showed that elevation in BMI led to the percentage of the Ff 'allele' becoming predominant, while the percentage of the FF 'allele' was in the normal BMI range. Also, BMI elevation caused significant reductions in VD and VDR expression, with significant elevations in alkaline phosphatase and the levels of calcium and phosphate in serum. Also, oxidative stress increases with increasing BMI. Elevation in BMI causes a reduction in VD concentration and VDR gene expression levels. Also, the percentage of heterozygous mutant genotype Ff 'allele' is predominantly in the obese human, in contrast to normal subjects, where the percentage of homozygous wild genotype FF 'allele' is predominant. In general, the genetic expression and polymorphism of VD and VDR can be used as a genetic marker for predisposition, diagnosis, prognosis, and progression of obesity.

## Introduction

Obesity reflects a long-term positive energy balance and excessive weight gain^1^. Obesity is most commonly caused by excess energy consumption (dietary intake) relative to energy expenditure (energy loss via metabolic and physical activity)^2^. There are several causes of obesity, including food, behavioral, environmental, drug, and genetic factors^3,4^. It has been estimated that genetic background can explain 40% or more of the variance in body mass in humans. The genetic component of human obesity is complex and likely to involve the interactions between multiple genes. Recent genome-wide association studies (GWAS) of height and the body mass index (BMI) in 250,000 European adults discovered 700 and 100 virtually independent single nucleotide polymorphisms (SNPs) related to these parameters, respectively^5^. BMI is a simple index of weight for height that is commonly used to Classify underweight, normal weight, overweight, and obesity in adults^6^. BMI is calculated as an individual’s weight in kilograms divided by the height in meters squared^7^. According to the World Health Organization, obesity is defined as a BMI of at least 30 kg/m^2^. There are three classes of obesity, Class 1: (BMI 30.00–34.99 kg/m^2^), class 2: (35.00–39.99 kg/m^2^), and class 3 (≥ 40.00 kg/m^2^). Overall excess of fat, usually defined by BMI, has long been recognized as a risk factor for metabolic-related diseases, such as cardiovascular diseases (CVD), type 2 diabetes mellitus (T2D), bone fragility as well as non-metabolic derangements such as non-alcoholic fatty liver disease (NAFLD)^8^**.** An estimated 205 million men and 297 million women over the age of 20 were obese—a total of more than half a billion adults worldwide. Worldwide, at least 2.8 million people die each year as a result of being overweight or obese. Excess weight during childhood and adolescence remains one of the most important issues in global health, despite emerging as a concern several decades ago^9,10^. Recent estimates suggest that 40 million children under the age of 5 years and more than 330 million children and adolescents aged 5–19 years were overweight or obese in 2016^11^^.^ Adipose tissue, especially in the visceral compartment, has been considered a simple energy depository tissue. Also, it acts as an active endocrine organ and releases a variety of biologically active molecules recognized as adipocytokines or adipokines. Excess intra-abdominal adipose tissue accumulation, often known as visceral obesity, is part of a phenotype that includes defective subcutaneous adipose tissue expansion and ectopic triglyceride (TG) storage, both of which are linked to a clustering of cardiometabolic risk factors. Among the many metabolic changes associated with this condition are hypertriglyceridemia, increased free fatty acid availability, adipose tissue release of proinflammatory cytokines, liver insulin resistance and inflammation, increased liver very low-density lipoprotein (V-LDL) synthesis and secretion, reduced clearance of TG-rich lipoproteins, the presence of small, dense LDL particles, and lower high-density lipoprotein (HDL) cholesterol levels. Age, gender, genetics, and ethnicity are all broad etiological factors that influence visceral adipose tissue accumulation. Sex hormones, local cortisol production in abdominal adipose cells, endocannabinoids, growth hormone, and dietary fructose may all play a role in proportionally enhanced visceral fat storage when faced with positive energy balance and weight gain. Physiological characteristics of abdominal adipose tissues such as adipocyte size and number, lipolytic responsiveness, lipid storage capacity, and inflammatory cytokine production are significant correlates and even possible determinants of the increased cardiometabolic risk associated with visceral obesity^1,7,8^.

Obesity is characterized by chronic low-grade inflammation with permanently increased oxidative stress^12,13^. Oxidative stress refers to a significant imbalance between ROS (and/or RNS) generation and antioxidant protection (in favor of the former), causing excessive oxidative damage^13–15^. Oxidative stress can be subcategorized into metabolic, drug-dependent, environmental, or nutritional oxidative stress. Over-expression of oxidative stress damages cellular structures along with the under-production of antioxidant mechanisms, resulting in the development of obesity-related complications^12,13,16^.

Vitamin D is a group of fat-soluble vitamins responsible for increasing intestinal absorption of calcium, phosphate, and magnesium, and many other biological effects^1–3^. In humans, vitamin D_3_ (cholecalciferol) and vitamin D_2_ (ergocalciferol) are the most important compounds in this group. The major natural source of VD is a synthesis of cholecalciferol in the lower layers of the epidermis of the skin, via a photo-chemical reaction of UVB light from sun exposure^1^. Cholecalciferol and ergocalciferol can be taken from the diet and supplements^1,2^. Only a few foods, for instance, the flesh of fatty fish, naturally contain valuable amounts of vitamin D^2,13^. Because sun exposure in the community varies and advice for the amount of sun exposure that is acceptable is uncertain in light of the danger of skin cancer, dietary guidelines normally presume that all of a person's vitamin D is taken by mouth^17,18^. It is assumed that most people need dietary vitamin D to reach the recommended serum level, i.e. greater than 30 ng/mL (75 nmol/L)^19^. The biological activity of vitamin D obtained through food or skin production is inactive. It is activated by two protein enzyme hydroxylation steps, the first in the liver and the second in the kidneys^2,3^. Ergocalciferol is metabolized to 25-hydroxyergocalciferol in the liver, while cholecalciferol is transformed to calcifediol (25-hydroxycholecalciferol). The serum levels of these two vitamin D metabolites, often known as 25-hydroxyvitamin D (VD), are used to assess a person's vitamin D status^17^. The kidneys and some immune system cells continue to hydroxylate calcifediol to create calcitriol (1,25-dihydroxycholecalciferol), the physiologically active form of VD. In the blood, calcitriol circulates as a hormone, plays a major role in the regulation of calcium and phosphate concentration, and promotes the healthy growth and remodeling of bone^1^. In addition, calcitriol has other impacts, involving some on cell growth, immune and neuromuscular functions, and decline of inflammation^3,8,12,13^. Active VD form exerts its effect through interaction with vitamin-D receptors (VDR)^17^. VDR belongs to the nuclear receptor superfamily of ligand-dependent transcriptional factors. After binding to its ligand, VDR interacts with the nuclear receptor retinoic acid X receptor (RXR). In the presence of vitamin D3, the VDR/RXR complex binds small sequences of DNA known as VD response elements and initiates a cascade of molecular interactions that modulate the transcription of a multitude of genes in tissues throughout the body^20^. The gene encoding VDR is located on chromosome 12cen-q12, contains 11 exons, and spans approximately 75 kilobases of genomic DNA. Allelic variants of the gene encoding VDR, recognized by *Apa*I (allele A/a), *Bsm*I (allele B/b), *FokI* (allele F/f) and *TaqI* (allele T/t) restriction endonucleases, The *FokI* polymorphism is a T/C transition polymorphism (ATG to ACG) at the first of two potential translation initiation sites in exon II, has been defined using the *FokI* restriction endonuclease^21^. The current study was done to evaluate the relationship between the elevation of BMI and the differentiation in VD level, VDR gene expression, and genetic polymorphism in adult Egyptian women. The study focused on the determination of VDR gene polymorphism by PCR–RFLP, VDR gene expression by qRT-PCR, and VD differentiation levels. Additionally, in the serum of adult Egyptian normal, overweight, and obese, the lipid profile, glucose level, alkaline phosphatase (ALP), calcium, phosphorus, and oxidative stress including, oxidant (malondialdehyde: MDA) and anti-oxidants (reduced glutathione: GSH and superoxide dismutase: SOD) were determined.

## Results

### Levels of VD and VDR expression in all studied groups

**Figure 1:**
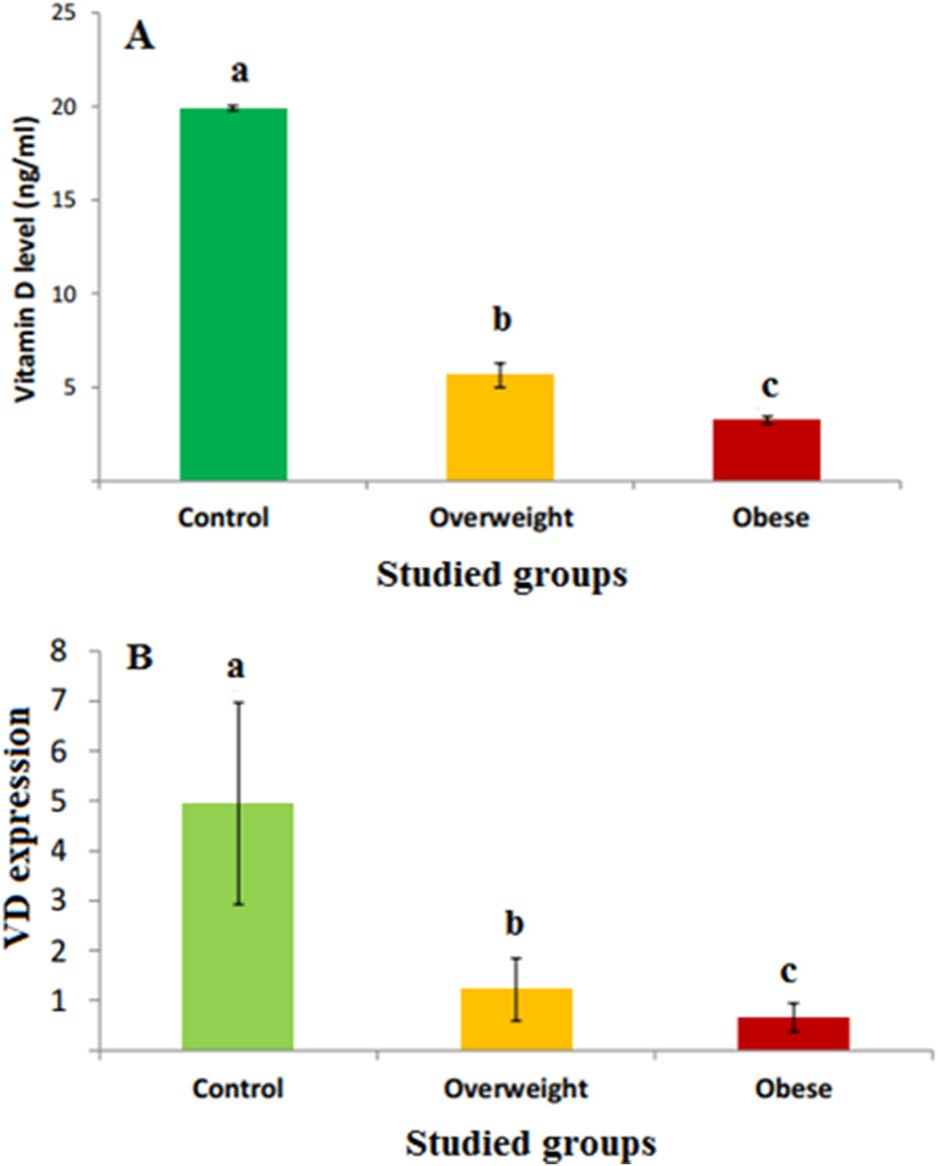
The levels of VD (**A**) and VDR expression (**B**) in different studied groups. The values are expressed as mean ± SE. Where the number of subjects in the control, overweight, and obese groups was 15, 25, and 30, respectively.

**Figure 2:**
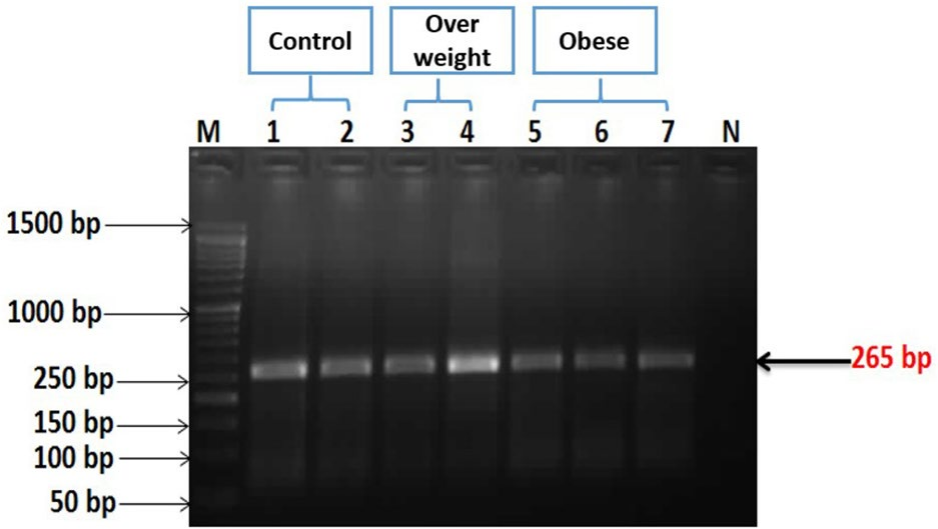
Agarose gel electrophoresis of VDR gene PCR products. Lane M is a DNA ladder (1.5 kb), lanes 1–7 are VDR PCR products (265 bp) and lane N is a negative sample.

**Figure 3:**
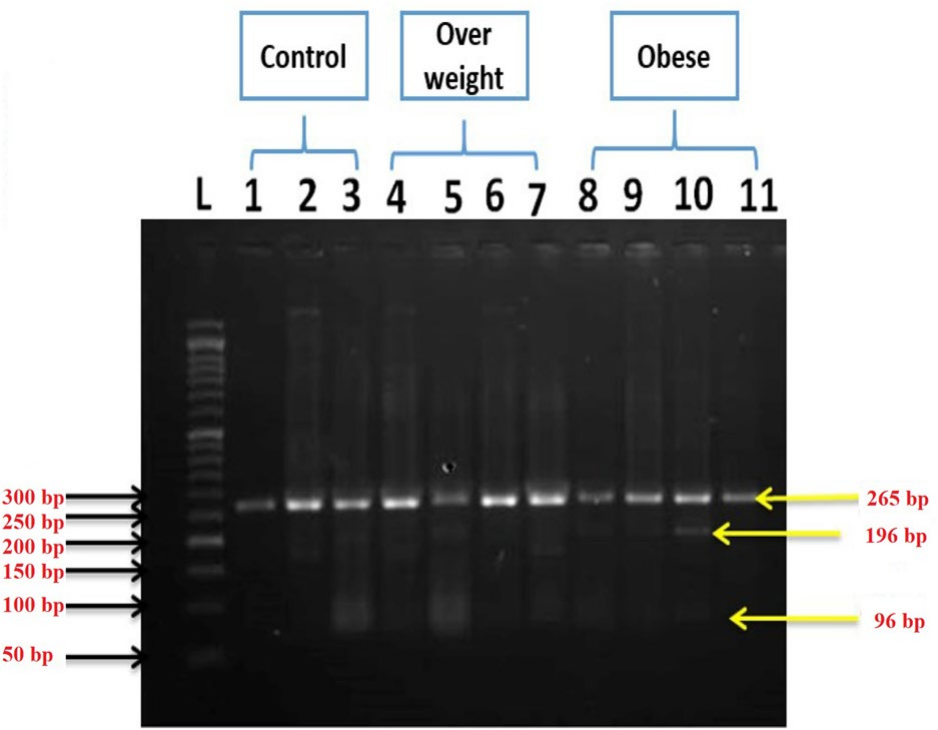
Restriction Endonuclease digestion for *FokI* polymorphism. FF indicates homozygous allele, FF: wild homozygous allele (265 bp), and Ff: heterozygous mutant (265 bp, 196 bp, 69 bp). L-DNA ladder.

Table 1 represents a comparison between the three studied groups (normal, overweight, and obese) according to demographic data. Figure 1A shows that VD levels in overweight and obese groups were decreased significantly (p ≤ 0.05) by about 71% and 83.6%, respectively, as compared with the normal group. Figure 1B shows that the levels of VDR expression were significantly decreased (p ≤ 0.05) in the overweight and obese groups by about 75.35% and 86.66%, respectively) as compared with the normal group. Figure 2 shows the VDR PCR product (265 bp) (see the supplementary figures a and b). Figure 3 shows the differences in VDR genetic polymorphism in the studied groups (see the supplementary figures c and d). Normally, the restriction endonuclease digestion for *FokI* polymorphism gives FF, Ff, and ff, indicating the presence of a wild homozygous allele, a heterozygous mutant, and a homozygous mutant (null allele), respectively. The results showed the presence of FF and Ff in all studied groups (normal, overweight, and obese) with different percentages. Where the percentage of homozygous wild genotype FF 'allele' was predominant in the normal group, followed by the overweight, and then obese groups, where the percentages were about 73.3, 40.0, and 33.3, respectively. In contrast, the percentage of heterozygous mutant genotype Ff 'allele' was predominant in the obese group, followed by the overweight, and then the normal groups, where their percentages were about 66.7, 60.0, and 26.7, respectively as shown in (Table 2).

**Table 1:** Comparison between all studied groups according to demographic data.

Variable	Control (n = 15)	Overweight (n = 25)	Obese (n = 30)	p
Age (years)	28.67 ± 0.85	33.12 ± 1.08	34.93 ± 1.33	0.006*
Weight (kg)	64.93 ± 1.85	80.20 ± 1.70	106.7 ± 2.58	< 0.001*
Height (m)	1.69 ± 0.02	1.68 ± 0.01	1.65 ± 0.02	0.123
BMI (kg/m^2^)	22.65 ± 0.34	27.64 ± 0.30	39.32 ± 1.17	< 0.001*

BMI: body mass index.

*Statistically significant at p ≤ 0.05.

**Table 2:** Comparison between the three studied groups according to Fok1.

p	χ^2^	Obese (n = 30)		Overweight (n = 25)		Control (n = 15)		Fok1
		%	No.	%	No.	%	No.	
0.034*	6.774*	33.3	10	40.0	10	73.3	11	FF
		66.7	20	60.0	15	26.7	4	Ff
		0.0	0	0.0	0	0.0	0	ff
		p_1_ = 0.041*, p_2_ = 0.011*, p_3_ = 0.609						Sig. bet. grps
0.125	4.158	66.7	40	70.0	35	86.7	26	F
		33.3	20	30.0	15	13.3	4	f

χ^2^: Chi-square test.

p: p-value for comparing between three groups and each of two groups.

p_1_: p-value for comparing between control and overweight.

p_2_: p-value for comparing between control and obese.

p_3_: p-value for comparing overweight and obese.

*Statistically significant at p ≤ 0.05.

### Calcium, phosphorus, and ALP levels

**Figure 4:**
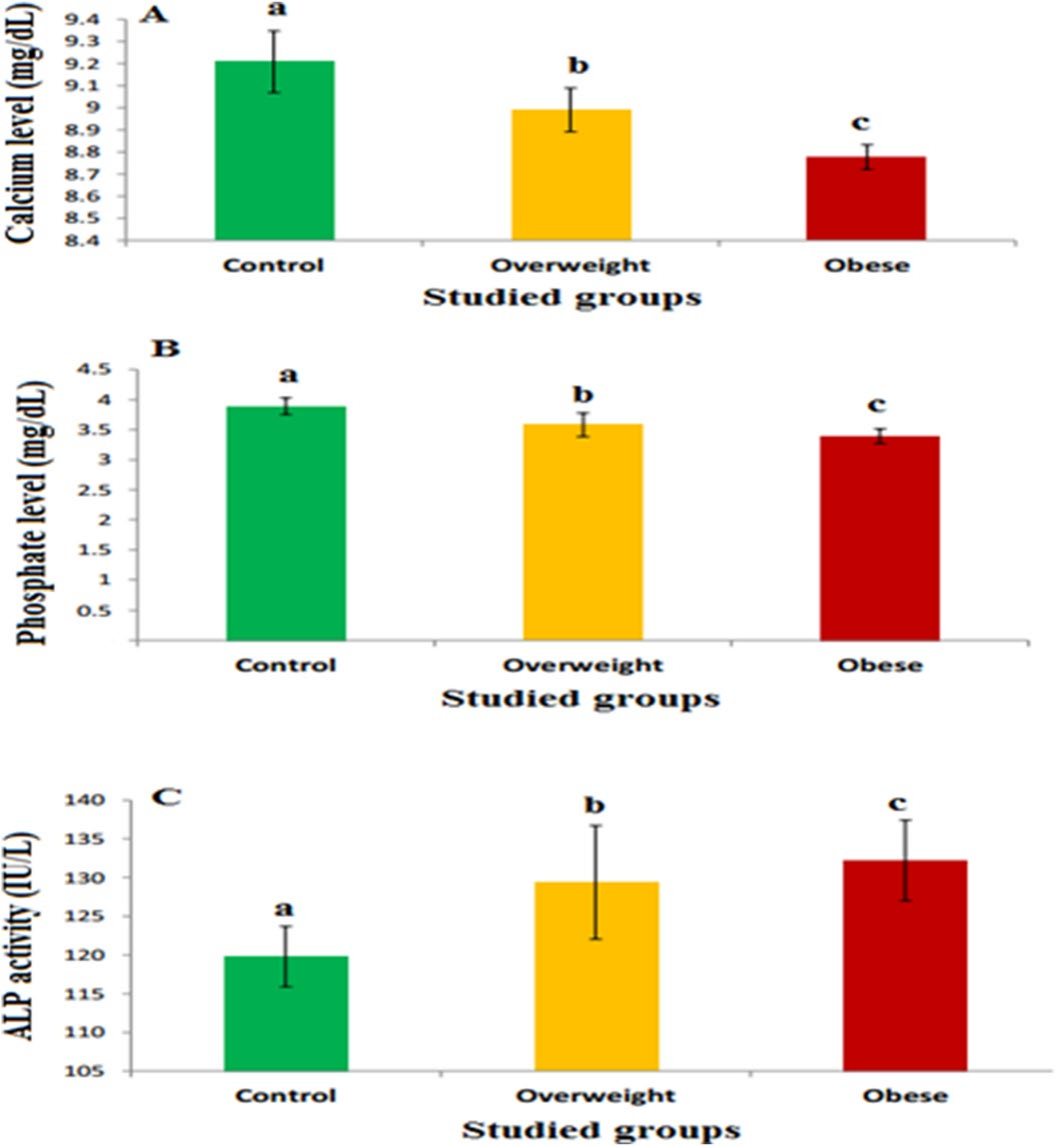
The levels of some minerals and alkaline phosphatase in different studied groups. (**A**) The calcium levels, (**B**) the phosphorous levels, and (**C**) the alkaline phosphatase levels. The values are expressed as mean ± SE. Different letters for the same parameter are significantly different at p ≤ 0.05 compared to the control. The number of subjects in the control, overweight, and obese groups was 15, 25, and 30, respectively.

The calcium levels in overweight and obese groups were decreased significantly by about 2.28% and 4.67%, respectively,as compared with the normal group (Fig. 4A). Also, the phosphorus levels were decreased in overweight (non-significantly, p > 0.05, by 8.01%) and in obese (significantly (P ≤ 0.05, by 12.84%) groups (Fig. 4B). However, the activity of ALP were increased in overweight [non-significantlyby 7.95% (p > 0.05)] and in obese [significantly by 10.12% (P ≤ 0.05)] groups as compared with the normal group (Fig. 4C).

### Glucose and lipid profile levels

Table 3 shows that the fasting blood glucose levels were increased in the overweight group (nonsignificantly: p > 0.05) and in the obese group (significantly: p ≤ 0.05) by about 3.17%, and 8.84%, respectively, as compared with the normal group. The levels of cholesterol, triglyceride, and LDL were increased significantly (P ≤ 0.05) in the overweight group (by about 30.15%, 59.47%, and 21.73%, respectively) and in the obese group (by about 63.94%, 10.31%, and 27.97%, respectively) as compared with the normal group Table 3. However, HDL levels were decreased (non-significantly, p > 0.05) in the overweight group and (significantly, P ≤ 0.05) in the obese group by about 13.27% and 31.91%, respectively, as compared with the normal group Table 3.

**Table 3:** Glucose and lipid profile levels in the serum of all studied groups.

Variable	Control (n = 15)	Overweight (n = 25)	Obese (n = 30)	p
Glucose (mg/dl)	79.57 ± 2.5	82.10 ± 3.46	86.61 ± 1.54	0.006*
TCC (mg/dl)	158.35 ± 4.0	206.10 ± 2.5	252.53 ± 5.3	< 0.001*
TG (mg/dl)	111.64 ± 2.7	135.90 ± 1.7	183.03 ± 2.3	0.123
LDL (mg/dl)	101.07 ± 3.2	111.50 ± 4.5	129.34 ± 2.4	< 0.001*
HDL (mg/dl)	44.28 ± 2.3	38.40 ± 2.0	30.15 ± 1.27	> 0.005

TCC: total cholesterol concentration; TG: triglycerides; LDL: low-density lipoproteins; HDL: high-density lipoproteins.

*Statistically significant at p ≤ 0.05.

### Levels of oxidant and antioxidant markers

**Figure 5:**
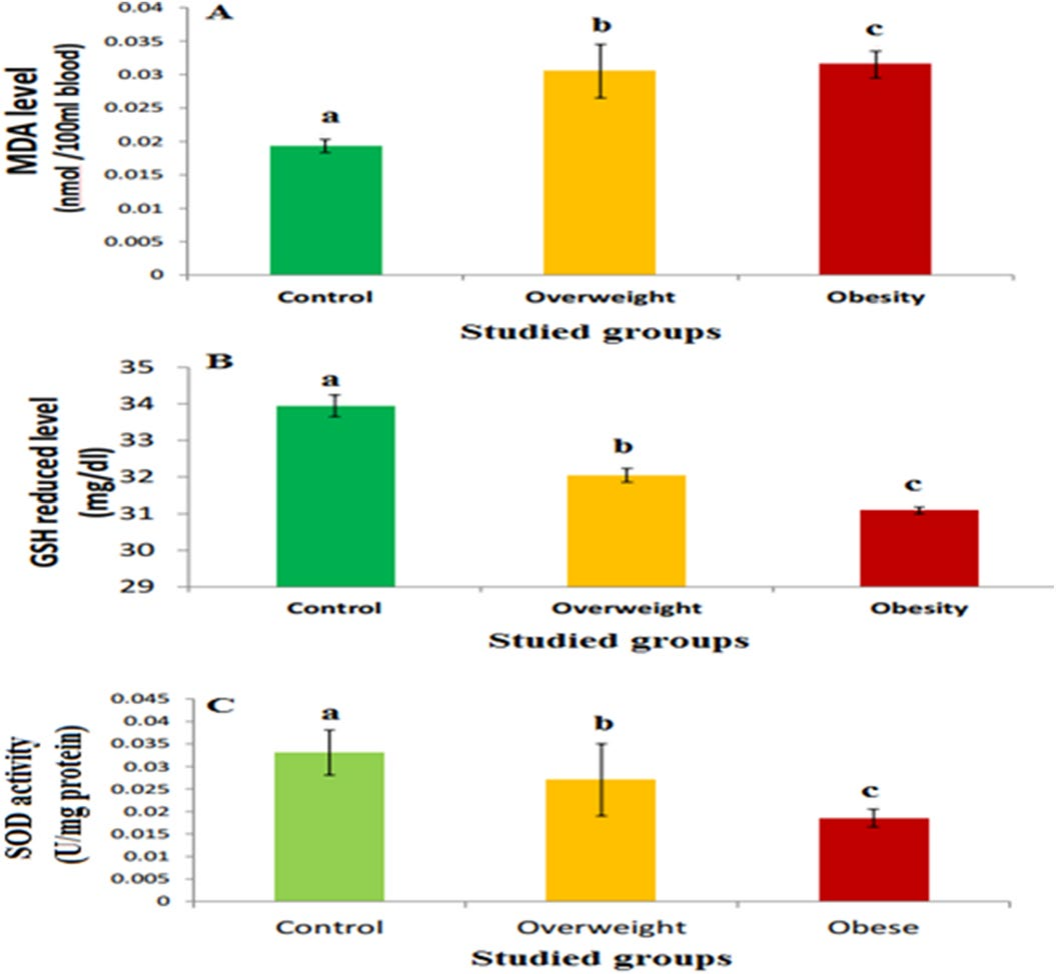
Oxidative stress in different studied groups. (**A**) The MDA levels, (**B**) the GSH levels, and (**C**) the SOD activities in different studied groups. The values are expressed as mean ± SE. Different letters for the same parameter are significantly different at p ≤ 0.05 compared to the control. The number of subjects in the control, overweight, and obese groups was 15, 25, and 30, respectively.

The results revealed that the levels of MDA, the end product of lipid peroxidation, were increased significantly (p ≤ 0.05) in overweight and obese groups by about 58% and 63.2%, respectively, as compared with the normal group. While GSH levels were decreased significantly (p ≤ 0.05) in the overweight and obese groups by about 5.59% and 8.39%, respectively, (Fig. 5A,B). Also, SOD activities were decreased significantly (p ≤ 0.05) in the overweight group and in the obese group by about 18.4% and 44.1%, respectively, as compared with the normal group (Fig. 5C).

## Discussion

Obesity develops when energy intake exceeds energy expenditure over a prolonged period of time, leading to an accumulation of body fat. Both environmental and genetic factors contribute to the development of obesity^22^. The previous study showed that there is a relationship between obesity and VD concentrations in human body. However, our study was carried out to evaluate the biochemical and molecular aspects of VD genetic polymorphism as a genetic indicator for obesity. This study focused on the determination of VD concentration, VDR genetic polymorphism, and VDR gene expression, ALP, calcium, and phosphorus. In addition, fasting blood glucose level, lipid profile, and oxidative stress, including oxidants such as MDA and antioxidants such as GSH and SOD were determined.

The results of the current study showed that the VD concentration in the blood was decreased with increasing BMI. Since VD is a fat-soluble vitamin, the reduction in VD concentration may be related to its accumulation in adipose tissues^23^**.** The active form of VD exerts its effect through interaction with VDR^16^**.** Our results revealed that VDR gene expression levels were decreased with increasing BMI and this reduction was followed the order: obese < overweight when compared with the normal. The reduction in VDR gene expression with the elevation of BMI is in harmony with the reduction of VD levels**.** It has been reported that anumber of polymorphisms in the VDR gene have the ability to change the activity of VDR protein^24^**.** In the current study, it had been detected the genetic polymorphism of *FokI* site by PCR–RFLP technique in all studied groups (normal, overweight and obese). Normally the restriction endonuclease digestion for *FokI* polymorphism gives: FF indicating wild homozygous allele; Ff indicating heterozygous mutant and ff indicating homozygous mutant (null allele). Our results illustrated the presence of FF and Ff in all studied groups (normal, overweight and obese) with different percentages. Additionally, the data revealed that thepercentage of homozygous wild genotype FF 'allele' was predominant in the normal group followed by the overweight group and then the obese group (73.3%, 40% and 33.3%, respectively). However, the percentage of heterozygous mutant genotype Ff 'allele' was predominant in the obese group followed by the overweight group and then the normal group (66.7%, 60% and 26.7%, respectively). This indicates that FF % was decreased with increasing BMI, while Ff % was increased and this points to that the elevation in BMI had a negative effect on the activity of VD. This conclusion is confirmed with the current results of VD level and VDR gene expression which exemplified that the elevation in BMI caused significant decreases in their levels. Moreover, the increase in BMI led to the mutation of *FokI* resulting in the reduction in FF% and elevation in Ff% alleles. Our results are in agreement with the previous study^21^, but disagree with the other study which was carried out on Indian women^25^**.**

Furthermore, the present results showed that the increases in BMI caused a significant reduction in calcium and phosphorus levels. This demonstrates that the reduction in VD concentration led to the reduction of the absorption of calcium and phosphorus from the intestine and their re-absorption from the kidney^26,27^. These results agree with the previous studies which reported that the levels of calcium and phosphorus are decreased with increasing BMI^28,29^**.** On the other hand, the present results showed that fasting blood glucose level was increased with increasing BMI. The elevation of glucose in the blood circulation may be related to the direct effect of dietary carbohydrate, and also it may be due to elevation of insulin resistance since insulin resistance is increased with increasing BMI^30^**.** The reduction in insulin level and elevation in insulin resistance leads to the reduction in glucose transporter 4 (GLUT-4)^30^ which is responsible for the facilitated diffusion of circulating glucose down its concentration gradient into fat cells and muscle^30^. Moreover, the present results illustrated that the increases in BMI led to raises of cholesterol, triglyceride and LDL levels, but decreased HDL level. The elevation in triglyceride may be due to the reduction of blood calcium where the reduction in calcium level in blood stimulates the biosynthesis of parathyroid hormone and 1,25-hydroxyvitamin D, which in turn elevate the intracellular calcium level leading to the activation of lipogenesis and inhibiting lipolysis^31^. The elevation of cholesterol, triglyceride, LDL and decreased of HDL lead to hypertension and arteriosclerosis resulting in cardiovascular diseases. These results agree with previous studies^29,32^**.** Moreover, the result showed that ALP activity was increased with increasing BMI and this may be due to the increases in the number or size of fat cells or a combination of both which depends on the degree of BMI^33^. Where previous studies reported that ALP activity is increased with increasing adipocytes and adipose tissues thatare considered as a source of serum ALP^34,35^.

On the other hand, the existing results demonstrated that MDA levels in the overweight and obese groups were significantly increased, while the GSH level and SOD activity were significantly decreased when compared with the normal group. This indicates that the elevation in the body weight induced oxidative stress and increased lipid peroxidation via enhancing the generation of free radicals such as superoxide radicals (O2^•-^) and hydrogen peroxide (H_2_O_2_). These free radicals increased the lipid peroxidation of polyunsaturated fatty acids in the membranes, and this led to the loss of cell integrity, increased the permeability of the membrane, and altered both inner membrane potential and calcium homeostasis, all of which resulted in cell death^13,36–41^. The resistance of many cells against oxidative stress is associated with high intracellular levels of GSH^42,43^. GSH exists in reduced (GSH) and oxidised (GSSG) forms^19^. The ratio of GSH to GSSG within cells is a measure of cellular oxidative stress. GSH protects cells from damage caused by reactive oxygen species, peroxides, lipid peroxides and free radicals, via neutralizing them as revealed in the following reactions: [2 GSH + R_2_O_2_ ⟶ ½ GSSG + 2 ROH, (where R = H or alkyl); and with free radicals GSH + R^٠^⟶½ GSSG + RH]. Also, GSH participates in thiol protection and redox regulation of cellular thiol proteins under oxidative stress by protein S-glutathionylation, a redox-regulated post-translational thiol modification as shown in the reaction: RSH + GSH + [O]⟶GSSR + H_2_O, where RSH is the protectable protein. Additionally, GSH is used in the detoxification of hazardous metabolites, such as methylglyoxal and formaldehyde, which are formed during oxidative stress. In addition, It maintains exogenous antioxidants for example vitamins E and C in their reduced forms.The decline in GSH level in the overweight and obese groups may possibly be due to increased its demand for lipid hydroperoxide metabolism by GPx to terminate free radical reactions^13,36–38,44–46^.

SOD is the first line of defense in the body against superoxide radicals, and it is considered the most effective antioxidant^13,36,39,47^. It catalyzes the dismutation (or partitioning) of the superoxide radical into molecular oxygen (O_2_) and hydrogen peroxide (H_2_O_2_). Superoxide is produced as a by-product of oxygen metabolism and, if not regulated, causes numerous types of cell damage^3^. Also, hydrogen peroxide is damaging and is degraded by other enzymes, such as catalase. So, SOD is an important antioxidant defense in almost all living cells exposed to oxygen. The inhibition of SOD activity in overweight and obese groups may be related to the action of superoxide radicals, in a free state or after their transformation to H_2_O_2_, with the active site of the enzyme or with its gene expression^13,14,37,38,41,48^.

Generally, our results demonstrated that the elevation in body mass index induced oxidative stress via the generation of free radicals and this can lead to various diseases such as cardiovasular disease. our results agree with the previous studies which showed that reactive oxygen species production increases exclusively in the adipose tissue of obese mice, accompanied by increasing expression of NADPH oxidase and decreasing expression of antioxidative enzymes^49,50^.

## Conclusion

The current study showed the following: VD concentration and VDR gene expression levels in the blood decreased with increasing BMI. Homozygous wild genotype FF and heterozygous mutant genotype Ff alleles were present in normal, overweight, and obese individuals with different percentages. Where the percentage of FF 'allele' was predominant in the normal group followed by the overweight group and then the obese group, while the percentage of Ff 'allele' was predominant in the obese group followed by the overweight group and then the normal group. Furthermore, oxidative stress increased with increasing body mass index, where its level in obese humans was greater than in overweight ones. In general, the genetic expression and polymorphism of VD and VDR can be used as a genetic marker for predisposition, diagnosis, prognosis, and progression of obesity.

## Material, subjects and methods

### Materials

Kits for the determination of alkaline phosphatase (ALP), calcium, and phosphorus were obtained from (BioSystems, USA). ELISA kit for assay of VD, Kits for blood DNA extraction, VDR gene amplification and expression, and VDR gene Polymorphism were obtained from (Qiagen GmbH, Hilden, Germany). 5,5′,dithiobis-2-nitrobenzoic acid, and GSH were acquired from (Sigma-Aldrich, St Louis, MO, USA). Thiobarbituric acid was gained from El-Nasr Pharmaceutical Chemicals Co. (Alex., Egypt). Kits for determination of total protein (TP), total cholesterol concentration (TCC), HDL, LDL and TG were gained from (Biodiagnostic, Cairo, Egypt).

### Subjects

#### Ethics approval

This study protocol was reviewed and approved by the Ethics Committee of the Faculty of Medicine, Alexandria University, under serial number 0106512. Since all subjects were collected after informed consent was obtained from all subjects and/or their legal guardian(s). All experiments were in conformity with the “Recommendations for Handling of Laboratory Humans for Medical Research” and complied with the Committee on Safety and Ethical Handling Regulations for Laboratory Experiments at ALEXU. Human studies were carried out following the ARRIVE guidelines and are in accordance with the 1964 Helsinki Declaration and its later modifications or comparable ethical standards^51^.

In the current study, seventy Egyptian subjects were classified into three groups harmonized by age and gender according to BMI into the normal BMI (BMI ≥ 18.5 and < 25 kg/m^2^), overweight (BMI ≥ 25 and < 30 kg/m^2^) and obese (BMI ≥ 30 kg/m^2^) groups (n = 15, n = 25 and n = 30, respectively) according to the WHO^52^. All Obese subjects were recorded from the obesity clinic at the Alexandria Main University Hospital, under supervision professor/Mohamed Nabil and Assistant Lecturer/Ahmed A. Abdul-Aziz (Endocrinology). It was confirmed that no subject included suffering from any chronic diseases such as diabetes mellitus or hypertension, renal, liver, or cardiac disease, and no medications or vitamin supplementation was taken. For each subject, a detailed history was taken and a comprehensive clinical examination was done. Also, a family history of obesity was recorded. Additionally, weight, height, waist circumference, and hip circumference were measured. Then BMI was calculated by dividing weight (kg) by height (m) squared. Blood samples from each subject was taken after overnight fasting 12 h by venipuncture in anti-coagulant EDTA for determination of the VDR as well as tubes for serum.

#### Biochemical assays

##### Determination of minerals, ALP, and VD

In serum, ALP, VD, and minerals including calcium and phosphorus were determined using kits^53,54^.

##### Glucose and lipid profile in serum

Fasting blood glucose and lipid profile include TCC, HDL, LDL and TG were determined using kits^55,56^.

##### Determination of oxidative stress in serum

MDA, the most abundant aldehyde end product of lipid peroxidation, was determined using thiobarbituric acid. Since one molecule of MDA reacts with two molecules of thiobarbituric acid to yield a pink chromagen which measures at 532 nm^57^. GSH as an antioxidant marker was assayed spectrophotometrically^58,59^ by the reaction of the sulfhydryl group of GSH with 5,5′-dithiobis-(2-nitrobenzoic acid) giving a yellow product measured at 412 nm and expressed as mg/mg protein. The activity of SOD was determined according to the method of Marklund and Marklund^60^. SOD activity is characterized as the enzyme quantity which inhibits the pyrogallol autoxidation rate through standard conditions, and the difference in the absorbance at 420 nm was measured in 2 min. SOD activity is expressed as U/mg protein.

##### Determination of VDR gene expression and VDR gene polymorphism

VDR gene expression and VDR gene polymorphism were determined using kits by qRT-PCR and PCR–RFLP, respectively. The blood samples collected in EDTA tubes were used in the extraction of DNA from peripheral blood leukocytes using the quick-DNA miniprep kit according to manufacturers’ instructions. Genomic DNA was amplified and analyzed for VDR genotypes by polymerase chain reaction (PCR) and restriction fragment length polymorphisms (RFLP). For determination of *FokI* genotypes, the forward and reverse primers in Table 4 were used. Where the PCR reaction components (25 µL of the mixture) was prepared by adding 12.5 µL of the master mix, 2 µL of the forward primer, 2 µL of the reverse primer, 1.5 µL of DNA and finally 7 µL of double distilled H_2_O.The PCR program was applied as follows, initial denaturation at 94 °C for 5 min, 34 cycles of 94 °C for 45 s, annealing at 60 °C for 45 s, and extension at 72 °C for 45 s, a final extension step at 72 °C for 3 min. PCR products were separated on agarose gel electrophoresis using 2% (w/v) agarose in 0.5 × of TBE buffer, the molecular size of the band was estimated using DNA molecular length marker (50 bp), finally, the gel was photographed by using gel documentation system. The PCR products were digested by incubation with the restriction enzyme, the amplicon was incubated with the *FokI* restriction enzyme at 37 °C for 5 min to get the three genotypes on 2.5% agarose gel designated FF, Ff, and ff. The molecular size (MS) of the FF polymorphic wild type homozygote exhibited 265 bp, while the MS of Ff heterozygote mutant exhibited 265 bp, 196 bp, and 96 bp. But, the MS of ff homozygote mutant exhibited 196 bp, and 96 bp fragments.Total RNA was extracted from the stored blood samples (at − 80 °C prior to extraction). The genomic RNA was isolated from blood samples by IQeasy plus blood RNA extraction mini kit according to manufacturers’ instructions. RNA concentration and purity were measured by Nanodrop. Senifast™ sybp^®^ low-redox one-step Kit had been used to quantify RNA in a single step assay after normalization to a certain concentration. Glyceraldehyde 3-phosphate dehydrogenase (GAPDH) was used as a reference gene for normalizing mRNA levels of the target genes and optimizes the integrity of the reaction. Oligonucleotide primers for GAPDH and VDR were recorded in (Table 4).

**Table 4:** Oligonucleotide primers sequences used for PCR-RFLP and qRT-PCR in this study.

Experiment	Primers	Primer sequence (5′ → 3′)	GC (%)	Tm (°C)	Ta (°C)	PCR product size
PCR-RFLP	FOK-1F	AGC TGC CCC TGG CAC TGA TCC TGC TCT	63	70.6	60	265 bp
	FOK-1R	ATG GAA ACA CCT TGC TTC TTC TCC CTC	48.1	60.9		
qRT-PCR	VDRqF	GAG GTG TCT GAA GCC TGG AG	60	58.1	60	–
	VDRqR	ACC TGC TTT CCT GGG TAG GT	55	58.6		
	GAPqF	AGC CCA GAA CAT CAT CCC TG	55	70.3	60	**–**
	GAPqR	CAC CAC CTT CTT GAT GTC ATC	47.6	68.8		

All qRT-PCRs were performed in duplicate. The qRT-PCR reaction was carried out in 20 μL into thermopickoqRT-PCR apparatus as described below in (Table 5). Delta Delta threshold cycle (ΔΔCT) expression value was calculated for RNA samples of each group for the determination of gene expressions of VDR according to the following equations:

$$\Delta {\text{CT }} = {\text{CT }}\left( {\text{a target gene VDR}} \right) \, - {\text{CT }}({\text{a reference}}\;{\text{gene GAPDH}}).$$

$$\Delta \Delta {\text{CT }} = \, \Delta {\text{CT }}\left( {\text{a target sample patients sample}} \right) \, - \Delta {\text{CT }}({\text{a reference}}\;{\text{sample}}\;{\text{normal}}\;{\text{sample}})$$

$${2}^{{ - \Delta \Delta {\text{Ct}}}} = {\text{ ratio of normalized expression}}$$

**Table 5:** 2-Step cycling qRT-PCR thermal cycling program.

Cycle	Temp (°C)	Time	Notes
1	45	10 min	Reverse transcription
1	95	2 min	Polymerase activation
40	95	5 s	Denaturation
	60	20 s	Annealing/extension (aquire at end of step)

#### Statistical analysis

The data were articulated as means ± standard error (SE). One-way analysis of difference (ANOVA) via (SPSS, 18.0 Software, 2011), and the individual assessment was acquired by Duncan's multiple range test (DMRT). Different letters for the same parameter are significantly different at p ≤ 0.05 compared to the control.

### Supplementary Information

Supplementary Figures.

## Data Availability

All data generated or analyzed during this study are included in this published article.
