# Supplementary material for: The association between body mass index elevation and differentiation in vitamin D receptor gene expression, genetic polymorphism, and oxidative stress in adult Egyptian individuals
Source: Sci Rep. 2023 Oct 17;13:17696. doi: 10.1038/s41598-023-44607-4 (PMC10582247; doi:10.1038/s41598-023-44607-4)

Supplementary Figure Legends

**Figures a and b. Agarose gel electrophoresis of VDR gene PCR products.** (a): lane M is a DNA ladder (1.5 kb), lanes 1-16 are VDR PCR products (265 bp), and lanes 17-18 are negative samples. (b): lane M is a DNA ladder (1.5 kb), lanes 1-7 are VDR PCR products (265 bp) and lanes 8-9 are negative samples.

**Figures c and d. Restriction endonuclease digestion for *FokI* polymorphism.** (c): Lane M is a DNA ladder (1.5 kb), lanes 1, 15 are uncut band (265 bp), lanes 3, 5, 10, 12, 14, 16, 19, 22, 23, and 25 are FF (wild homozygous allele: 265 bp), lanes 2, 4,( 6 -9), 11, 13, 17, 18, 20, 21, and 24 are Ff (heterozygous mutant: 265 bp, 196 bp and 96 bp), and lanes 26-29 are negative results. (d): Lane M is a DNA ladder (1.5 kb), lane 1 is the uncut band (265 bp), lanes 2, 4, 6, 8, 11 are FF ( wild homozygous allele: 265 bp), lanes 3, 5, 7, 9,10 are Ff ( heterozygous mutant: 265 bp, 196 bp and 96 bp), and lanes 12-15 are negative results.


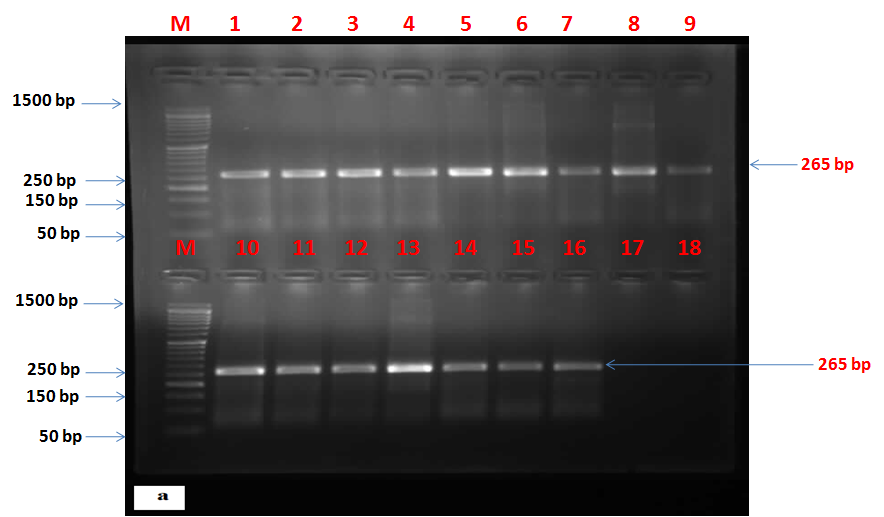


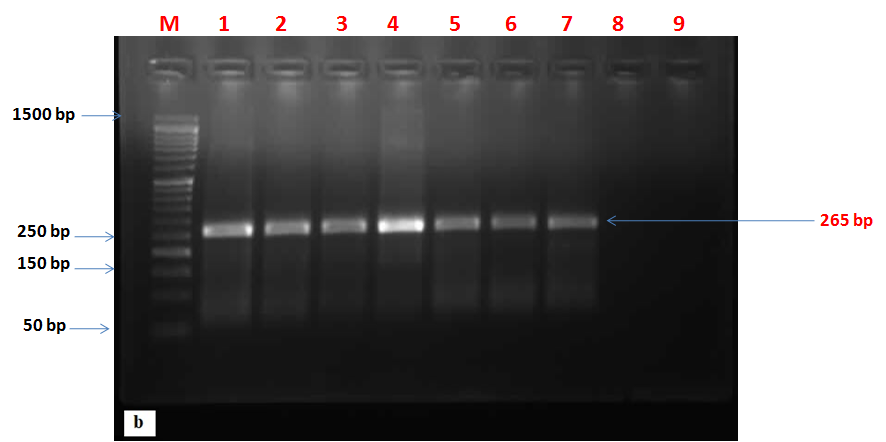


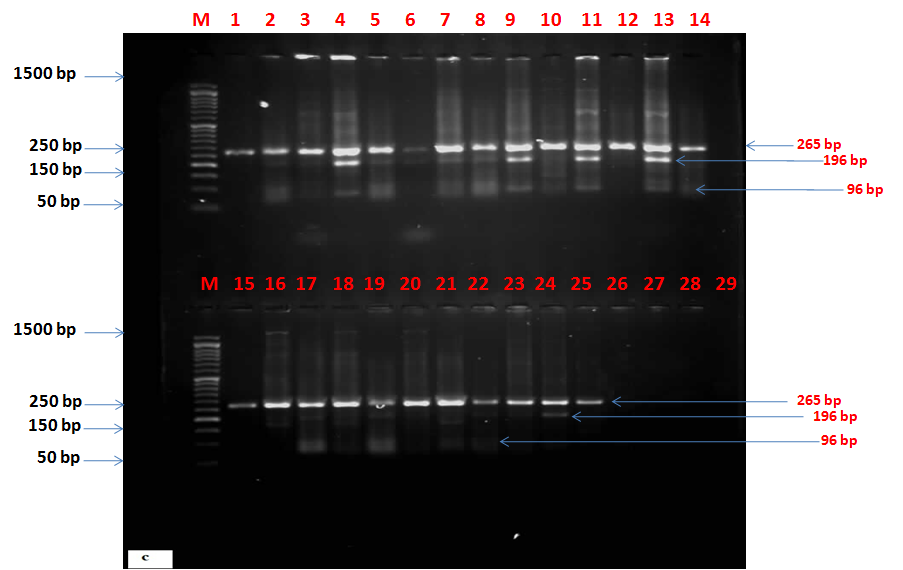


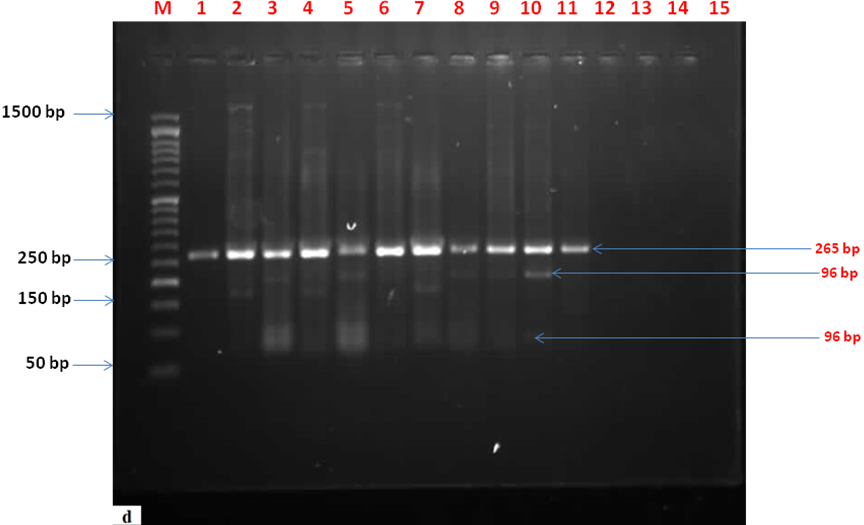

Supplement: Supplementary file 1 — Supplementary Figures. [file 41598_2023_44607_MOESM1_ESM.doc]
